# Ecofriendly metal-free olefins epoxidation and alcohol oxidation by *in situ* generated poly(peroxybenzoic acid) as a heterogeneous recyclable catalyst under mild conditions: an in-depth mechanistic study

**DOI:** 10.1039/d5ra00578g

**Published:** 2025-02-25

**Authors:** Boshra Mahmoudi, Milad Kazemnejadi

**Affiliations:** a Department of Dentistry, Tishk International University Sulaymaniyah Iraq Boshra.abbas@tiu.edu.iq; b Chemistry Department, Faculty of Sciences, Golestan University Gorgan Iran m.kazemnejadi@gu.ac.ir

## Abstract

In this study a mild and selective protocol was introduced for the alcohol oxidation and alkene epoxidation as two vital reactions in organic synthesis. Poly(benzoic acid) (PBA), a synthetic polymer, was used for the preparation of highly active poly(peroxybenzoic acid) (PPBA) *in situ* in the presence of H_2_O_2_. At ambient temperature, alcohol (primary) oxidation proceeds to carboxylic acid selectively (81–99% selectivity), and in 0 °C, aldehyde was the selective product (90–98% selectivity). Alkene epoxidation was also performed in 0 °C with high selectivity. The catalyst was compatible towards a wide variety of alcohols and alkenes substrates like acid sensitive substrates. PBA is used in the reaction at a catalytic rate and, in the presence of H_2_O_2_, which provides the oxygen necessary for oxidation, is converted to catalytically active PPBA species and reintroduced into the catalytic cycle. The mechanism of the oxidation and epoxidation processes was studied deeply. PBA could be recycled for several times without loss of catalytic activity.

## Abstract 2

A mild, metal free and selective method has been developed for the alcohol oxidation and olefin epoxidation using PBA/H_2_O_2_ catalytic system.

## Graphical abstract

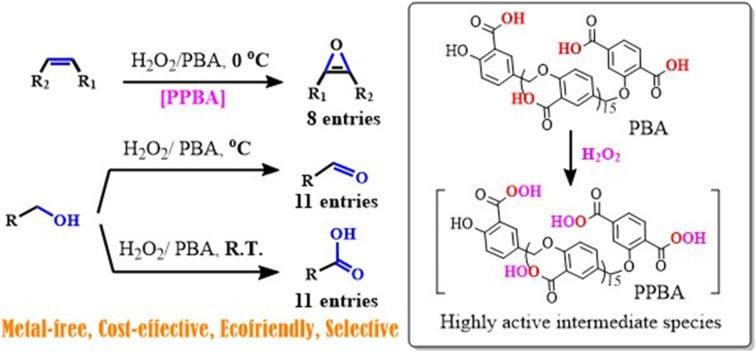

## 1. Introduction

The oxidation of alcohols and the epoxidation of alkenes are among the most widely used reactions in organic synthesis. Epoxides are one of the most reactive compounds in organic chemistry that has a wide application in the chemical industry, such as ethylene oxide and propylene oxide, which are widely used to prepare solvents, plasticizers and antifreeze.^1^ Epichlorohydrin is also a very important intermediate in the chemical industry, which is formed from the epoxidation of allyl chloride and is used in the preparation of epoxy resins, adhesives and coatings and composites. The epoxidation of vegetable oils as a plasticizer for PVC and the preparation of resins are also among the other applications of epoxidation in the chemical industry.^1,2^ Alkenes are also valuable precursors for the preparation of a variety of polymers by metallocene catalysts.^3–6^

The oxidation of alcohols, on the other hand, has a special place in the synthesis of pharmaceutical compounds due to the preparation of aldehyde, ketone and carboxylic acid products. Aldehydes and ketones are among the vital intermediates in pharmaceuticals, fragrances and polymers, and carboxylic acids are also widely used in the preparation of soaps, detergents and various organic acids that are used in the pharmaceutical and food industries.^7,8^

This wide application highlights its importance in terms of economics, large-scale production, environmental issues and selectivity. Metal-free catalysts fulfill all the aforementioned conditions and advantages compared to catalysts containing coordinated transition metals. A major problem of coordinated transition metal-based catalysts is metal leaching, which not only causes environmental pollution, but also reduces the efficiency of the catalyst in successive cycles and becomes practically inactive after several consecutive cycles, because the catalytically active sites are limited to the coordinated metals (and not all of them). In addition, transition metals are expensive and toxic, limiting their large-scale application.^9–11^ Furthermore, due to the diversity of oxidation and epoxidation products, the selective catalyst must have high selectivity. Low selectivity not only makes purification of the desired product challenging, but also makes it uneconomical at a larger scale.^12^ Therefore, the design of a catalyst system without a transition metal is important from various aspects.

Various catalytic systems have been reported for the metal-free oxidation of alcohols. TEMPO is one of the most widely used reagents in the field of metal-free oxidation of alcohols, which has high compatibility with a wide range of substrates (various types of primary and secondary alcohols). TEMPO-mediated alcohol oxidation under O_2_ (TEMPO/HCl/HNO_3_/O_2_;^13^ TEMPO/HBr/*t*-butyl nitrile/O_2_ (ref. 10)), and air (or aerobic) (TEMPO/Br_2_/NaNO_2_/air;^9^ TEMPO/HCl/NaNO_2_/air;^14^ TEMPO/NH_4_NO_3_/H^+^/air^11^) conditions, has demonstrated its ability to oxidize alcohols to the corresponding carbonyls.

Furthermore, nitrogen doped activated carbon^15^ and graphene nanosheets^16^ as well as B/P Co doped nanoporous carbon^17^ were applied for the aerobic oxidation of alcohols. The use of electrochemical methods^18^ and metal-free photocatalysts^19,20^ as well as (H)NO_*x*_ species (in the presence of O_2_, under Three-Phase Flow Conditions^21^) has also been developed for metal-free oxidation.

On the other hand, limited reports are available on the development of metal-free catalytic systems for the epoxidation of olefins. Previously, a transition metal-free alumina catalyst was developed for epoxidation of *cis*-cyclooctene with H_2_O_2_ 70% wt, where in the catalyst needs several steps for synthesis.^22^ Later, Lin *et al.*, introduced a nitrogen-doped onion-like carbon (OLC) for styrene epoxidation with higher catalytic activity than some other metal-related catalytic systems.^23^ In another study, cumene was served as a precursor for the *in situ* preparation of peroxy radical for the aerobic oxidation of olefins.^24^ This system could be promoted for gram scale synthesis, and air was applied as the oxidant and cumene could be recycled. Also, polydioxirane (PDOX) was also another metal-free active catalyst has been developed for olefin epoxidation.^25^

Recently, Kazemnejadi *et al.* developed a metal free approach for the selective olefine epoxidation by TAIm[X] (X = WO_4_^2−^, HSO_5_^−^) ionic liquid as a sustainable and recyclable catalyst.^26^ Despite extensive progress in the methods developed for the oxidation and epoxidation of alcohols and olefins, these systems have drawbacks such as being uneconomical (especially due to the use of transition metals and the design of complex and large catalytic systems), not being biocompatible, cumbersome and multi-step synthesis methods, *etc.*

**Scheme 1:**
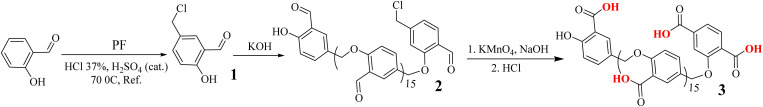
Synthesis of PSA and PBA.^23^

**Scheme 2:**
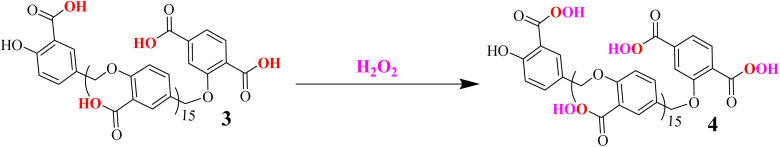
*In situ* preparation of (poly(peroxy benzoic acid)) in the presence of PBA/H_2_O_2_.

In this study, olefin epoxidation and alcohol oxidation were performed using PBA as a metal-free catalytic system in the presence of H_2_O_2_. Polybenzoic acid (PBA) is a synthetic polymer with multiple acid groups on the polymer chain (as pendant groups) (Scheme 1).^27^ PBA in the presence of H_2_O_2_ is converted to the highly active intermediate PPBA, which performs the aforementioned transformations under mild conditions (Scheme 2). The ability to oxidize alcohols in the presence of peracid (a mixture of organic acid and oxidant) has been demonstrated.^28–30^ The advantage of this method is its high selectivity, such that the selective oxidation of alcohols to aldehydes and selective oxidation to acids were carried out at 0 and 26 °C (ambient temperature), respectively. The selective epoxidation of alkenes was also carried out at 0 °C.

## 2. Experimental

### 2.1. Materials and instrumentations

All alcohols and alkenes substrates were provided from Merck company with 97–99% purity. The substrates were used without any further purification. The solvents were dried before use under N_2_ atmosphere. The progression of reactions (oxidations and epoxidations) was monitored through thin layer chromatography (TLC) on silica gel plates (polygram SILG/UV 254) and gas chromatography (GC) using a Shimadzu-14B GC with an HP-1 capillary column, nitrogen as the carrier gas, and anisole as an internal standard. FTIR (Fourier-transform infrared) analyses were obtained on a Perkin Elmer-RX1 Spectrometer.

### 2.2. Synthesis of PBA

PBA was synthesized according to our previous report (Scheme 1).^27^ Briefly, in first, poly salicylaldehyde (PSA) was synthesized by chloromethylation of salicylaldehyde 
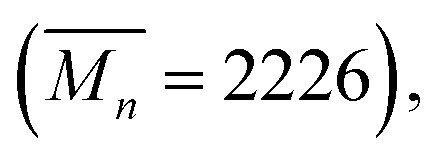
 reported elsewhere.^31–35^ In the second step, 0.1 g of PSA was added to 20 mL of distilled water in a 50 mL round-bottom flask equipped with a condenser. Then, 1.0 mmol of KOH and 1.0 mmol of KMnO_4_ was added to the balloon. The mixture was heated to 70 °C, and stirred for four hours. Finally, the resulting PBA was separated from the mixture by a simple filtration and treated with HCl (1.0 N) several times, followed by washing and dying in an oven at 50 °C (MW = 2396 g mol^−1^ (GPC analysis)^27^).

### 2.3. A general procedure for the epoxidation of olefins and selective primary-alcohol oxidation to aldehyde by PBA/H_2_O_2_

A small glass tube (with screw cap) was charged with alkene (2.0 mmol) and H_2_O_2_ 35% (0.2 mL) and then immersed in an ice-bath to complete cooling. Then, 0.5 g of PBA was added to the mixture and the mixture was stirred (using a small magnet bar) for appropriate time. The % conversion and % selectivity was monitored and recorded by GC or TLC (if possible).

After reaction completion, 1.5 mL of ethyl acetate was added to the mixture and, PBA was separated by simple filtration. The filtered PBA was washed with deionized water, dried in vacuum oven and stored for the next use. PBA could be stored in room temperature for several months. Alcohol oxidation follows a similar procedure, with 2.0 mmol of alcohol substrate added to the tube in the first step.

### 2.4. A general procedure for the direct oxidation of alcohols to carboxylic acids by PBA/H_2_O_2_

Direct and selective oxidation of primary alcohols to carboxylic acids were conducted at room temperature (26 °C) with a similar procedure described at alcohol oxidation.

## 3. Results and discussion

The number of acidic groups in PBA was measured by titration. In this experiment, 0.5 g of PBA was titrated with 1 M sodium hydroxide in the presence of phenolphthalein reagent. This experiment was repeated 3 times and the results for the number of acidic groups in PBA were obtained as 3.6, 3.7, 3.7 mmol/0.5. The results are in complete agreement with the molecular mass obtained by GPC analysis,^25^ based on which the degree of polymerization was calculated to be 15.

### 3.1. Oxidation/epoxidation parameters

All reactions were carried out in hydrogen peroxide (both as a solvent and as a reagent). Studies showed that the PBA : H_2_O_2_ ratio and temperature are two effective parameters in oxidation and epoxidation reactions. In order to study these parameters, the epoxidation reaction of styrene was chosen as a model reaction to investigate these two parameters. Studies showed that at 0 °C, the highest efficiency and selectivity for epoxystyrene equal to 98% (4 hours) was achieved. In the studies, a mechanism will be shown that increasing the temperature causes the formation of benzoic acid radicals on peroxybenzoic acid and changes the reaction path, in a way that causes the selectivity to decrease at temperatures above 50 °C. The oxidation reaction of alcohols at 0 °C was also effective for the conversion of benzyl alcohol to benzaldehyde and produced conversion and selectivity of 97% and 98%, respectively, for 2 hours.

Increasing the temperature for the oxidation of benzyl alcohols led to the selective product benzoic acid *via* a radical mechanism (Section 3.3). Unlike the epoxidation reaction, which lost selectivity with increasing temperature, the oxidation of benzyl alcohol at room temperature (26 °C) produced 99% selectivity to benzoic acid (99% conversion) for 1 hour. The radical nature of this mechanism was proven in studies.

**Fig. 1:**
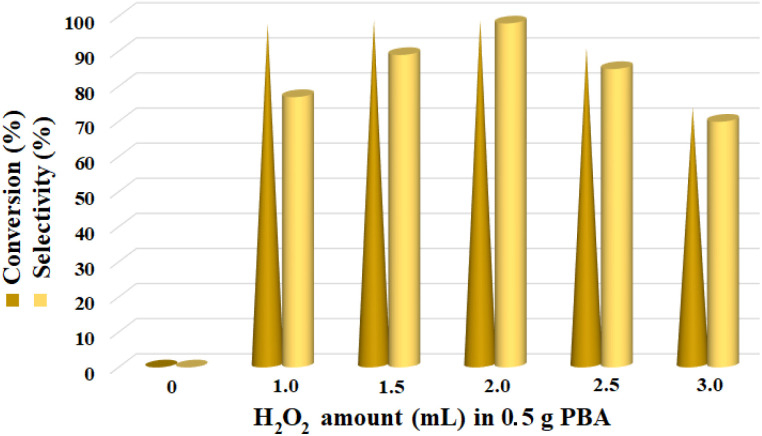
Screening of H_2_O_2_ : PBA (PSA amount was considered constant as 0.5 g) ratio over the epoxidation reaction of styrene at 0 °C for 4 h.

The second effective parameter, the H_2_O_2_ : PBA ratio, was also studied with different values (Fig. 1). The highest efficiency was obtained at the ratio of 2 mL/0.5 g for all reactions. Reducing the amount of hydrogen peroxide to 1.5 mL and 1 mL, respectively, led to a decrease in the conversion percentage to 89% and 77% (the selectivity was almost unchanged). Also, increasing hydrogen peroxide (to 25 and 3.0 mL) decreases the % conversion and % selectivity. Previous reports have shown that in epoxidation reactions in the presence of excess hydrogen peroxide, the epoxide ring is converted to the vicinal diols, thus reducing the efficiency (Fig. 1).^36^ At 2.5 mL of hydrogen peroxide, the conversion and selectivity percentages were calculated to be %85 and %90, respectively; at 3 mL of hydrogen peroxide, these values were %70 and %72, respectively. Increasing the hydrogen peroxide also increased the dilution of the solution, which had a negative effect on this reaction. The reaction did not produce any product in the absence of hydrogen peroxide, reflecting its critical role in the activation of polybenzoic acid and converting it to polyperoxybenzoic acid (the amount of PSA was 0.5 g in all reactions) (Fig. 1).

In order to demonstrate the performance of PPBA, in the control reaction of styrene epoxidation, the reaction was carried out in the absence of PBA. The results showed that PBA alone also had no observable catalytic performance towards alcohol oxidation and olefin epoxidation, reflecting that it is converted to catalytically active species in the reaction pathway in the presence of H_2_O_2_.

### 3.2. Catalytic activity

After achieving the best conditions for oxidation/epoxidation reactions, various alkene derivatives were studied for epoxidation in the presence of H_2_O_2_/PBA. Table 1 shows the epoxidation of various aliphatic and aromatic alkenes with H_2_O_2_/PBA. According to the results, all derivatives had good to excellent efficiency (in terms of % selectivity and % conversion) and the epoxidation was carried out in the time range of 2.5–6 h. Another advantage of this method was the selective epoxidation of acid-sensitive alkenes such as 14c, 14d, 14f–14h, which are well epoxidized in this method. In addition, the high selectivity of the method for dienes such as 14i, 14f, and 14j also allows for the selective epoxidation of only one group. Although compounds 14f and 14j are symmetrical, in compound 14i only the non-double double bond undergoes epoxidation (quite selectively).

**Table 1:** Metal free epoxidation of alkenes by H_2_O_2_/PBA catalytic system^a^

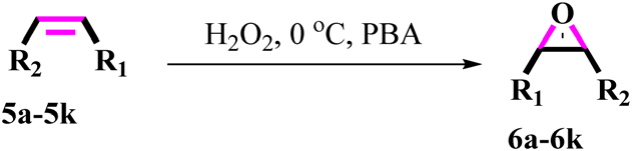							
Entry	Alkene (a)		Product (b)		Time (h)	Conversion^b^ (%)	Selectivity^b^ (%)
1	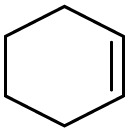	5a	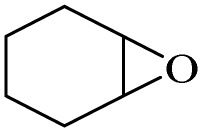	6a	3	95	98
2	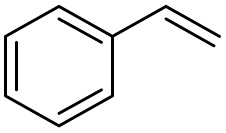	5b	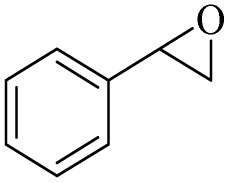	6b	4	98	98
3	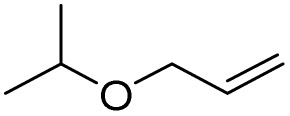	5c	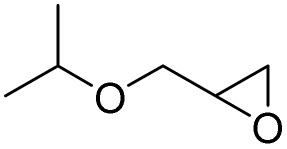	6c	5.5	90	96
4	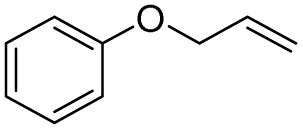	5d	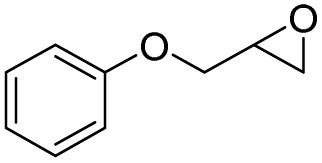	6d	2.5	98	98
5	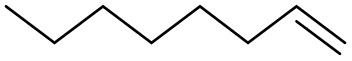	5e	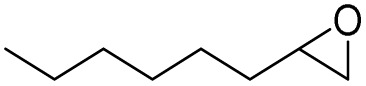	6e	6	90	95
6	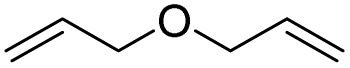	5f	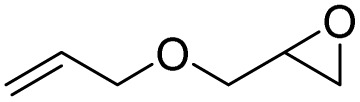	6f	4.5	90	96
7	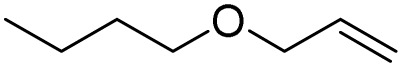	5g	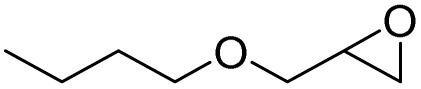	6g	5	92	95
8	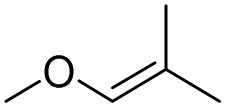	5h	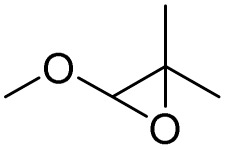	6h	5.5	90	97
9	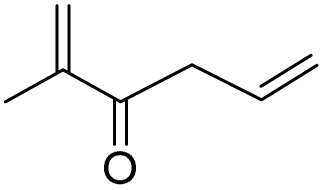	5i	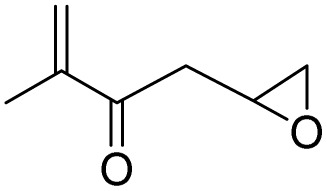	6i	4	96	96
10	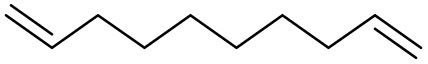	5j	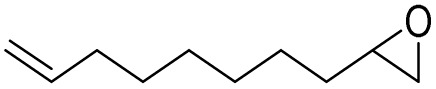	6j	6	90	95
11	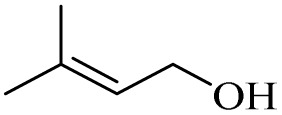	5k	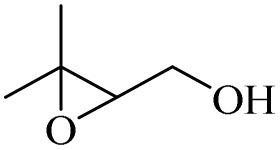	6k	4	96	98

a Reaction conditions: alkene (2.0 mmol), H_2_O_2_ 35% (0.2 mL), PBA (0.5 g), 0 °C (ice bath).

b Based on GC. Internal standard = anisole.

Next, the oxidation of various primary alcohols was studied at two different conditions (1) 0 °C and (2) room temperature (26 °C) by the H_2_O_2_/PBA catalytic system, and the results are summarized in Table 2.

**Table 2:** Selective oxidation of primary alcohols by H_2_O_2_/PBA catalytic system^a^

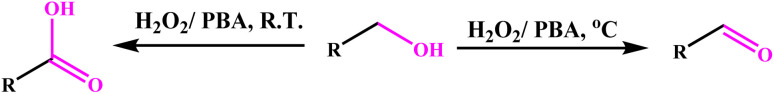							
Entry	Alcohol	Time (min)		Conversion^b^ (%)		Selectivity^b^ (%)	
		Aldehyde	Acid	Aldehyde	Acid	Aldehyde	Acid
1	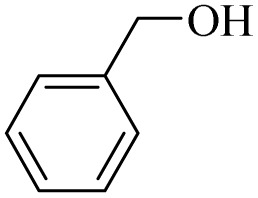	120	60	97	99	98	99
2	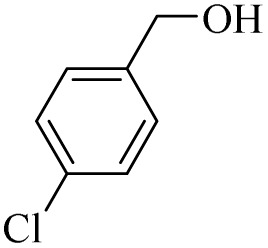	180	50	92	98	98	99
3	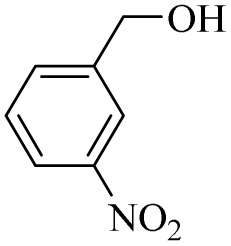	180	65	92	96	99	99
4	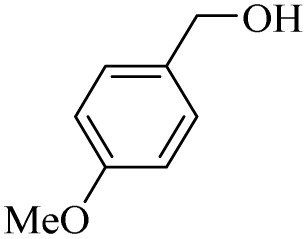	90	40	95	95	99	99
5	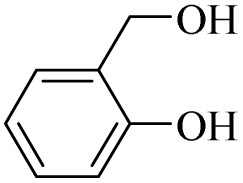	100	80	96	98	98	98
6	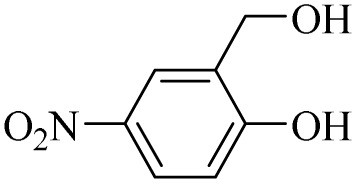	120	100	98	96	99	98
7	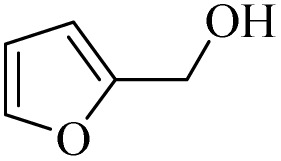	180	120	90	80	98	97
8	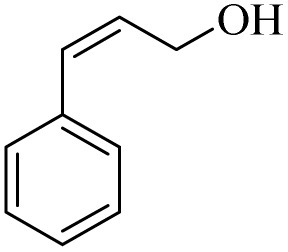	150	100	98	99	98	81
9	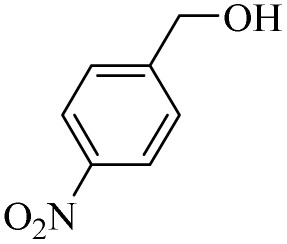	160	60	93	96	99	99

a Reaction conditions: primary alcohol (2.0 mmol), H_2_O_2_ 35% (0.2 mL), PBA (0.5 g), R.T. for aldehyde; 0 °C for carboxylic acid.

b Based on GC. Internal standard = anisole.

The difference in the observed products as well as the time, % conversion and % selectivity at these two temperatures was due to their different mechanisms, which are discussed in the next section. At room temperature, alcohols undergo selective oxidation to aldehydes at 0 °C, while at room temperature, the aldehyde is directly converted to carboxylic acids *via* a radical mechanism. Under radical conditions, the reaction times were in the range of 60–1210 min, while for the oxidation of alcohols to benzaldehyde at 0 °C, a time range of 90–180 was observed. Selectivities for all derivatives were in the range of 97–99% (except for cinnamyl alcohol). Also, no dominant electron effect due to electron-withdrawing and electron-donating substitutions was observed on the conversion percentage and selectivity.

The catalytic ability of H_2_O_2_/PBA for the conversion of secondary alcohols to carbonyls was also studied (Table 3). 1-Phenylethan-1-ol, cyclohexanol and 1,2,3,4-Tetrahydronaphthalen-1-ol were converted to the corresponding ketones at two temperatures: 0 °C and 26 °C (room temperature). The results of this table also provide further evidence of the mechanistic difference in these two methods. For example, acetophenone was converted to acetophenone at 0 °C in 240 minutes with a conversion of 85%, while at room temperature the time was reduced to 60 minutes and 90% conversion was observed. Similar results were obtained for the other two derivatives.

**Table 3:** Selective oxidation of secondary alcohols to ketones by H_2_O_2_/PBA catalytic system^a^

Entry	Alcohol	Time (min)		Conversion^b^ (%)		Selectivity^b^ (%)	
		At 0 °C	At 26^b^ °C	At 0 °C	At 26^b^ °C	At 0 °C	At 26^b^ °C
1	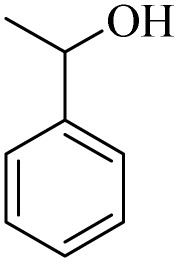	240	60	85	90	99	99
2	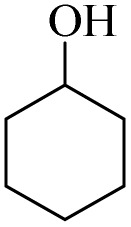	260	50	80	92	99	99
3	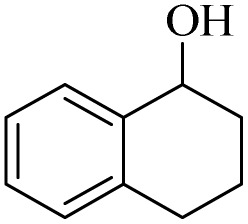	240	60	88	95	99	99
4	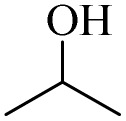	280	90	54	66	99	99

a Reaction conditions: alcohol (2.0 mmol), H_2_O_2_ 35% (0.2 mL), PBA (0.5 g). Based on GC. Internal standard = anisole.

b Radical mechanism.

### 3.3. Mechanism studies

**Scheme 3:**
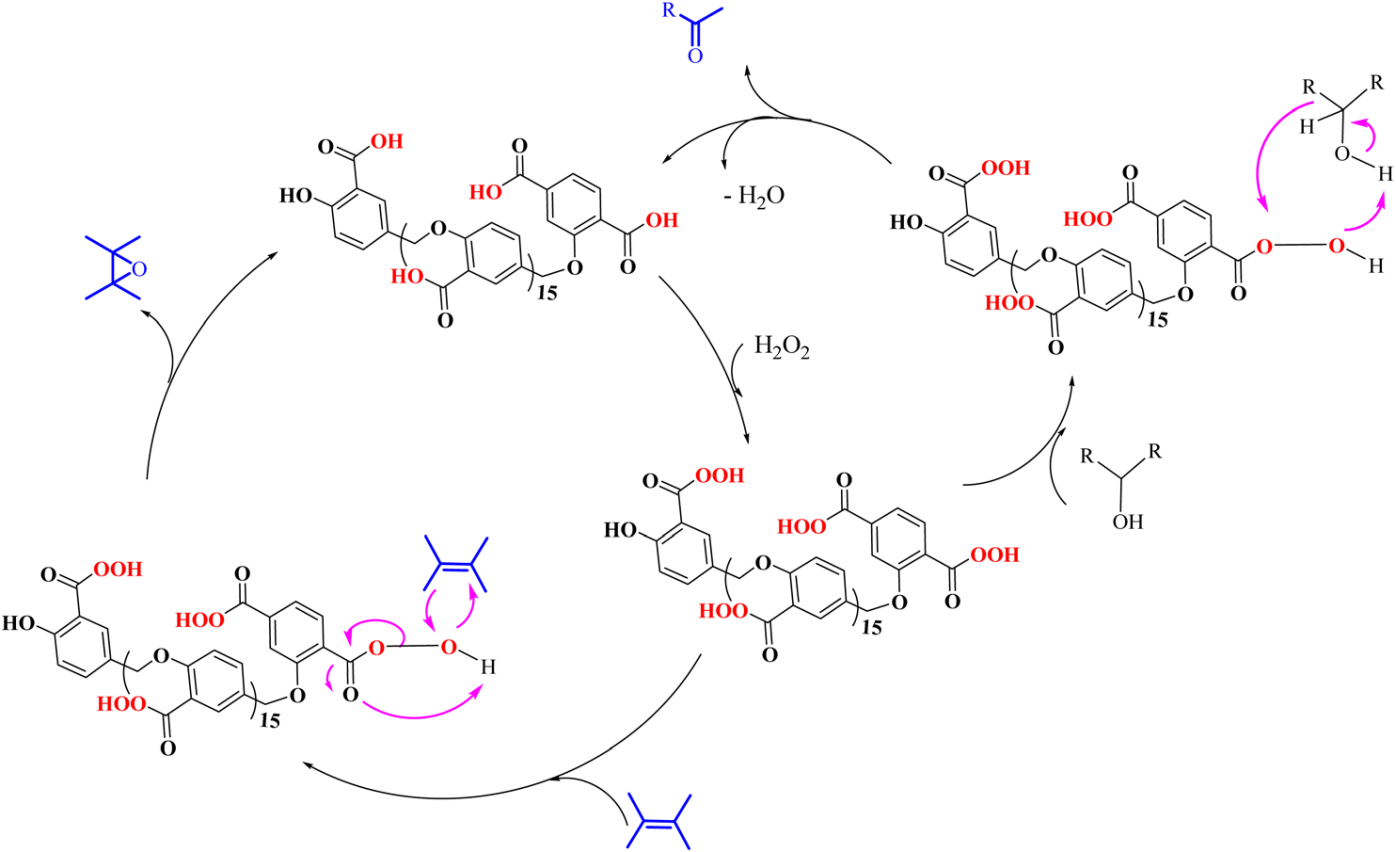
Two plausible mechanisms for the selective alcohol oxidation to aldehyde and olefin epoxidation by PBA (0 °C in an ice bath)—a none radical mechanism.

Scheme 3 shows the proposed mechanism for the epoxidation of olefins and the oxidation of alcohols in the presence of PBA/H_2_O_2_. As shown in Scheme 3, the reaction proceeds in two phases. One explanation for the reaction occurring in two-phase conditions is the structure of PBA and PPBA, which have both hydrophilic and hydrophobic groups and therefore act as a surfactant, reducing the surface tension of the aryl, resulting in better interaction of the catalyst with the alkyl (or alkyls). Examples of such behavior have also been observed for catalysts containing other hydrophilic and hydrophobic groups.^37,38^

PPBA could not be isolated and characterized from the environment due to its high reactivity. Previous reports have shown that a peroxybenzoic acid is not stable in aqueous media and that the hydroxy radical formed during the decomposition of peroxybenzoic acid is highly reactive^39,40^ and rapidly reacts with other molecules present in the reaction medium, and this high reactivity leads to its short lifetime in water.^40,41^ According to this proposed mechanism, PBA is initially converted to PPBA in the presence of H_2_O_2_. Radical formation from peroxybenzoic acid typically does not require an external initiator because peroxy acids can decompose spontaneously to form radicals. The mechanism involves the homolytic cleavage of the O–O bond in the peroxybenzoic acid molecule.^39–41^

H_2_O_2_ acts as an activator of polybenzoic acid by converting it to peroxybenzoic acid. Strong evidence for this conversion was the lack of epoxidation reaction in the presence of H_2_O_2_ and in the absence of PBA (Fig. 1). In the second step, oxygen is transferred from the peroxide active groups on PPBA to the olefin, forming the epoxide product and PBA returning to the catalytic cycle. The presence of hydrophilic acid and peroxyacid groups on PBA justifies the reaction in water solvent. Although initially a two-phase environment is created, as shown in the mechanism, the olefin approaches the PBA surface together with water, ultimately leading to epoxidation.

The oxidation of alcohols also passes through the PPBA intermediate. As shown in the cycle on the right (Scheme 3), the transfer of oxygen to the alcohol group occurs by the absorption of a proton from the carbon carrying the alcohol group by PPBA. Finally, the aldehyde product is obtained and PBA returns to the catalytic cycle. Under these conditions, further oxidation to benzoic acid does not occur. Optimization studies showed that with increasing the hydrogen peroxide ratio, the selectivity towards aldehyde decreases and leads to other products, including benzoic acid. In addition, in another experiment, the oxidation of aldehyde was studied at 0 °C and room temperature. Under these conditions, no products were observed at 0 °C, while at room temperature, almost similar results were obtained as in Table 4 and the % conversion for benzaldehyde and 4-methoxybenzaldehyde were calculated to be 98% and 96%, respectively, within 15 minutes.

**Table 4:** Oxidation of aldehydes to carboxylic acids by H_2_O_2_/PBA catalytic system^a^

Entry	Aldehyde	Time (min)		Conversion^b^ (%)		Selectivity^b^ (%)	
		At 0 °C	At 26 °C	At 0 °C	At 26 °C	At 0 °C	At 26 °C
1	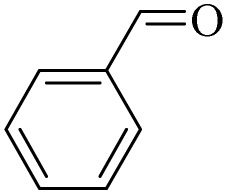	0	15	0	98	0	99
2	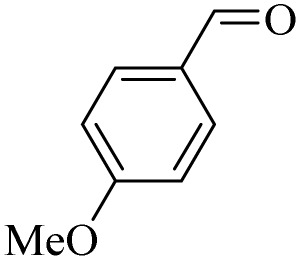	0	15	0	96	0	99

a Reaction conditions: aldehyde (2.0 mmol), H_2_O_2_ 35% (0.2 mL), PBA (0.5 g).

b Based on GC. Internal standard = anisole.

The observations confirmed the absence of any radical intermediates in the reaction mixture of alcohol oxidation as well as alkene epoxidation at 0 °C. For this purpose, in the control reaction of styrene epoxidation (0 °C), hydroquinone (0.3 g) was used as a radical scavenger at the beginning of the reaction.

The reaction progress was completely similar to before (absence of radical scavenger), indicating the absence of radical intermediates in the reaction mixture. Similar results were also obtained for the model reaction of oxidation of benzyl alcohol to benzaldehyde, in which the addition of 0.3 g (3 mmol) of hydroquinone had no effect on the conversion and selectivity.

**Scheme 4:**
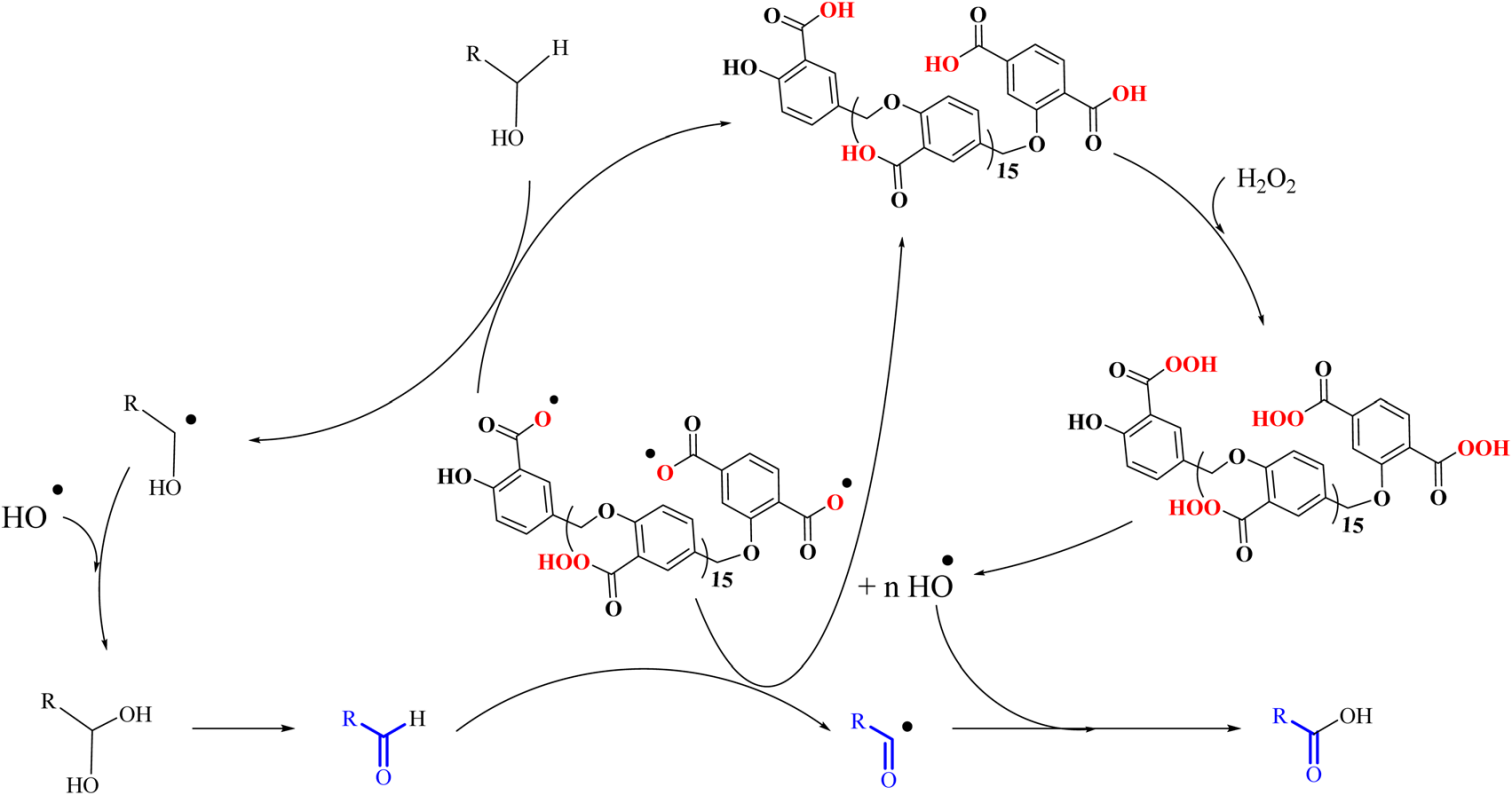
A plausible mechanism for direct oxidation of alcohols to benzoic acid by PBA in H_2_O_2_ in water at ambient temperature—a radical mechanism.

On the other hand, a radical mechanism for the direct oxidation of alcohols to benzoic acid was proposed based on the evidence obtained in this study including radical scavenger, direct conversion of alcohol to acid, butyl alcohol oxidation, ice-water bath test, cinnamyl alcohol oxidation, efficiency difference compared to °C (Scheme 4). The evidence indicated the presence of radical species at room temperature and in aqueous solvent. Scheme 4 shows the scheme of this proposed mechanism that initially, as in the previous mechanism, PBA is converted to the active PPBA species in the presence of H_2_O_2_. At room temperature and under reaction conditions (alcohol (2.0 mmol), H_2_O_2_ 35% (0.2 mL), PBA (0.5 g), H_2_O, R.T.), PPBA decomposes into the active radical species Ar–C(=O)–O˙ and HO˙. Benzyl hydrogens are converted to stable benzyl radicals in contact with the active Ar–C(=O)–O˙ species, which are then rapidly converted to hydrates in the presence of HO˙ radical species. By removing water, an aldehyde intermediate is formed, which is again converted to the active Ar–C=O˙ radical (derived from PPBA) in contact with the active radical species Ar–C(=O)–O˙. In the presence of HO˙ radical in the environment, it is converted to benzoic acid (the final product). The radical active species Ar–C(=O)–O˙ also return to the catalytic cycle in two steps, the first after the formation of the benzyl radical and the second after the formation of the active radical Ar–C=O˙.

**Fig. 2:**
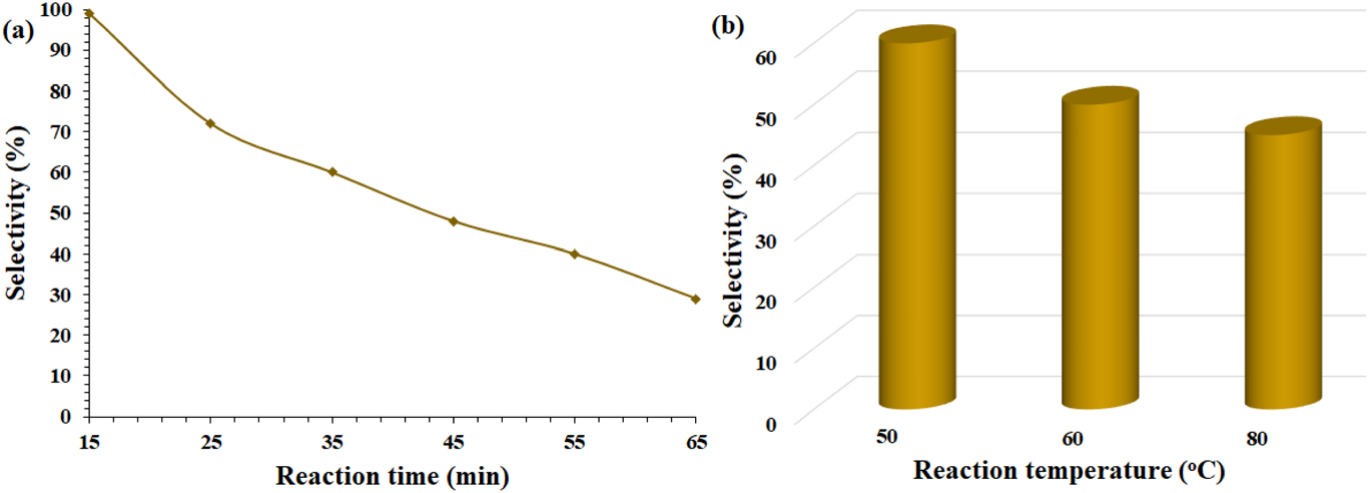
Selectivity monitoring over the epoxidation of styrene towards (a) reaction time after 15 min in an ice bath and (b) in different reaction temperature.

For this radical mechanism, several lines of evidence are available to prove its radical nature. (1) Use of radical scavenger: in order to prove the presence of radical species, hydroquinone was added as a radical scavenger at the beginning of the oxidation reaction of benzyl alcohol. The % conversion for this experiment after 1 h was about 7% (GC analysis). Compared with the oxidation at 0 °C where the addition of hydroquinone had no effect on the reaction, this difference in the result can be directly attributed to the presence of radical species in the reaction mixture, which is in agreement with previous reports.^42,43^ (2) The direct conversion of alcohol to acid is another evidence that strengthens the proposed radical mechanism. According to the proposed mechanism, the active radical Ar–C=O˙ is easily formed in the presence of radical species and undergoes subsequent reactions,^44,45^ and the aldehyde is an intermediate in this mechanism. (3) In another experiment, *n*-butyl alcohol was used as the initial substrate. Under completely similar conditions, the conversion percentage for butanoic acid was calculated to be 12%. The formation of the aliphatic radical is more difficult than the benzyl radical and requires a higher activation energy.^46^ The result of this experiment confirms the presence of benzyl radical species. (4) In another experiment, the flask containing the benzyl alcohol oxidation reaction mixture was transferred to an ice-water bath after 15 minutes, and the selectivity for benzoic acid was monitored every 10 minutes by GC. Fig. 2a shows the monitoring of this reaction. The selectivity for benzoic acid was calculated to be 99% in the first 45 minutes, and after being placed in the ice-water bath, it decreased sharply, reaching about 30% after one hour. These results reflect the fact that the reaction in the ice-water bath proceeds by another route that does not lead to carboxylic acid, and although the production of radical species is stopped, radical reactions are still taking place. However, the failure of the reaction to stop indicates the formation of other products. (5) In another experiment, the effect of temperature on the oxidation reaction of benzyl alcohol was studied. In this experiment, which was repeated three times at three temperatures of 50, 60 and 80 °C, the selectivity decreased to 60%, 50% and 45%, respectively (Fig. 2b). The % conversion were measured in the range of %98–99 for these three reactions. Radical intermediates undergo various side reactions at high temperatures^47^ and therefore selectivity was lost with increasing temperature. Similar results were also obtained in the study of Sankar *et al.*^48^

(6) Further evidence for a radical mechanism came from the low selectivity of cinnamyl alcohol at room temperature. As shown in Table 2, the selectivity of cinnamyl alcohol at room temperature decreases to 81% (to cinnamic acid), while the selectivity for all derivatives studied was reported to be in the range of 97–99%. This decrease in selectivity can be directly attributed to the presence of the olefinic bond in cinnamyl alcohol, which can participate in radical reactions and form by-products.

(7) In accordance with the results observed in Tables 2 and 3, the observed time courses for the oxidation of alcohols at 0 °C and room temperature differ significantly, reflecting the different oxidation pathways at the two different temperatures. This time difference was even more pronounced for the oxidation of secondary alcohols (Table 3).

**Scheme 5:**
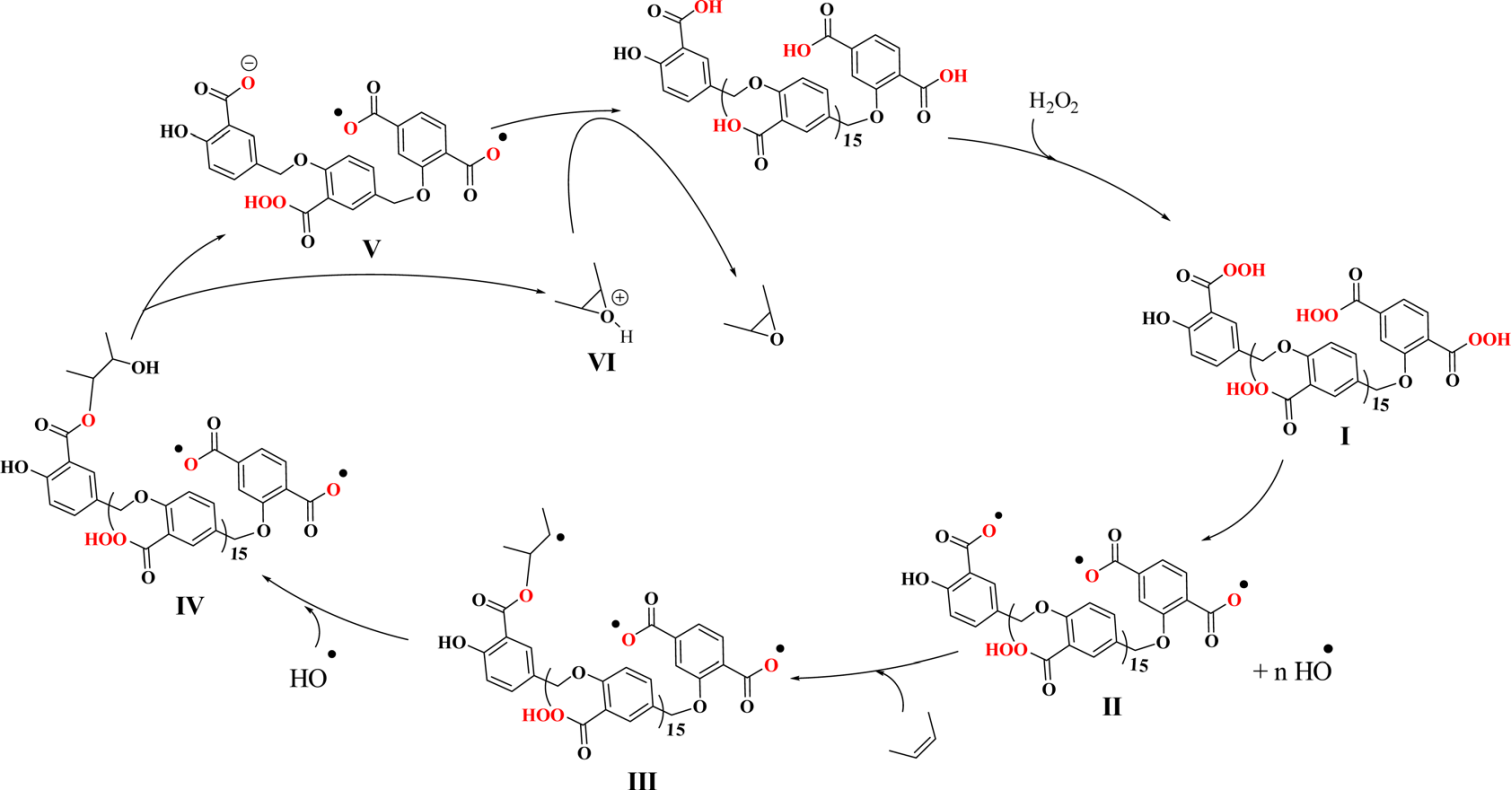
A proposed mechanism for the radical transformation of olefin to epoxide catalyzed by PBA/H_2_O_2_ system.

In contrast to the high selectivity observed for the direct oxidation of benzyl alcohol to benzoic acid under radical conditions, the epoxidation of olefins at room temperature did not show high selectivity. Scheme 5 shows a proposed mechanism for this transformation in the presence of radical intermediates. This mechanism was proposed based on the low selectivity observed for the epoxide product. Initially, and similar to previous mechanisms, the radical-active species Ar–C(=O)–O˙ (intermediate II, derived from PPBA) is formed in the presence of H_2_O_2_. The olefin reacts on the surface of this radical-active species to form intermediate III, which subsequently reacts with a hydroxyl radical to form the oxonium (VI) intermediate. Finally, by accepting a proton from the V anion, PBA returns to the catalytic cycle and forms the epoxide product. Intermediate III is a highly active species for any radical reaction, including reaction with another alkene and formation of new radical species. In addition, the reaction with RCOO˙ radical groups on PSA (in intermediates II–V) can also generate side products by reacting with the benzyl radical.

### 3.4. Recyclability tests for PBA

**Fig. 3:**
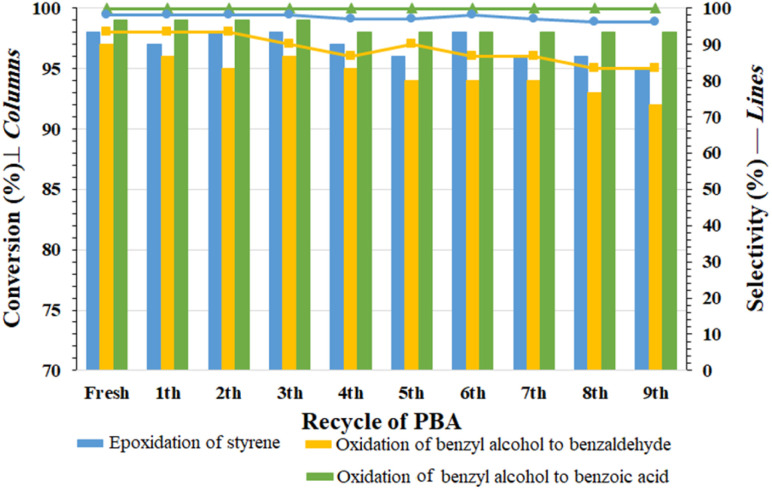
Recyclability of PBA towards the three reactions of (i) (Blue) epoxidation of styrene to epoxy styrene (reaction conditions: styrene (2.0 mmol), H_2_O_2_ 35% (0.2 mL), PBA (0.5 g), 0 °C, 4 h); (ii) (Yellow) selective oxidation of benzyl alcohol to benzaldehyde (reaction conditions: styrene (2.0 mmol), H_2_O_2_ 35% (0.2 mL), PBA (0.5 g), 0 °C, 2 h); and (iii) (Green) direct oxidation of benzyl alcohol to benzoic acid (alcohol (2.0 mmol), H_2_O_2_ 35% (0.2 mL), PBA (0.5 g), H_2_O, R.T.).

The recovered PBA from the reaction mixture was washed several times with distilled water and reused after drying in oven (50 °C). This cycle was repeated for 9 consecutive times for the reactions (1) styrene epoxidation, (2) benzyl alcohol oxidation to benzaldehyde and (3) direct oxidation of benzyl alcohol to benzaldehyde. The results are summarized in Fig. 3. The results shown in Fig. 3 reflect the recyclability and stability of PBA under oxidizing conditions and in the presence of H_2_O_2_. No significant decrease in efficiency or selectivity was observed in any of the oxidation and epoxidation reactions. This is an advantage over transition metal-based catalytic systems, which suffer from metal leaching in each cycle. In addition, the proposed system is simple and cost-effective, making it suitable for practical and industrial purposes.

In order to investigate the structure of the recovered PBA, the amount of carboxylic acid groups was measured by titration test after recovery cycles 2, 4, 7 and 9 in the three mentioned reactions (each test was repeated 3 times). The results are summarized in Table 5, and accordingly, the amount of carboxylic acid groups in these three cycles in the oxidation reaction of alcohol to benzoic acid was measured to be 3.6 (2nd), 3.8 (4th), 3.7 (7th), and 3.8 (9th) mmol/0.5 g of PBA, respectively (Table 5). A similar trend was also observed for the other two reactions. The results were quite similar to the freshly prepared sample and indicate the stability of PBA under the reaction conditions. These results also showed that the acidic groups in PBA are continuously converted to PPBA and return to PBA after the oxidation (or epoxidation) reaction.

**Table 5:** Determination of –CO_2_H groups in recycled PBA at three oxidation/epoxidation reactions by acid/base titration^a^

Number of cycles	mmol of –CO_2_H groups in 0.5 g of PBA		
	Reaction i	Reaction ii	Reaction iii
2nd	3.5	3.7	3.6
4th	3.6	3.7	3.8
7th	3.8	3.8	3.7
9th	3.6	3.7	3.8

a For the reaction conditions of i, ii, and iii see Fig. 3 caption.

**Fig. 4:**
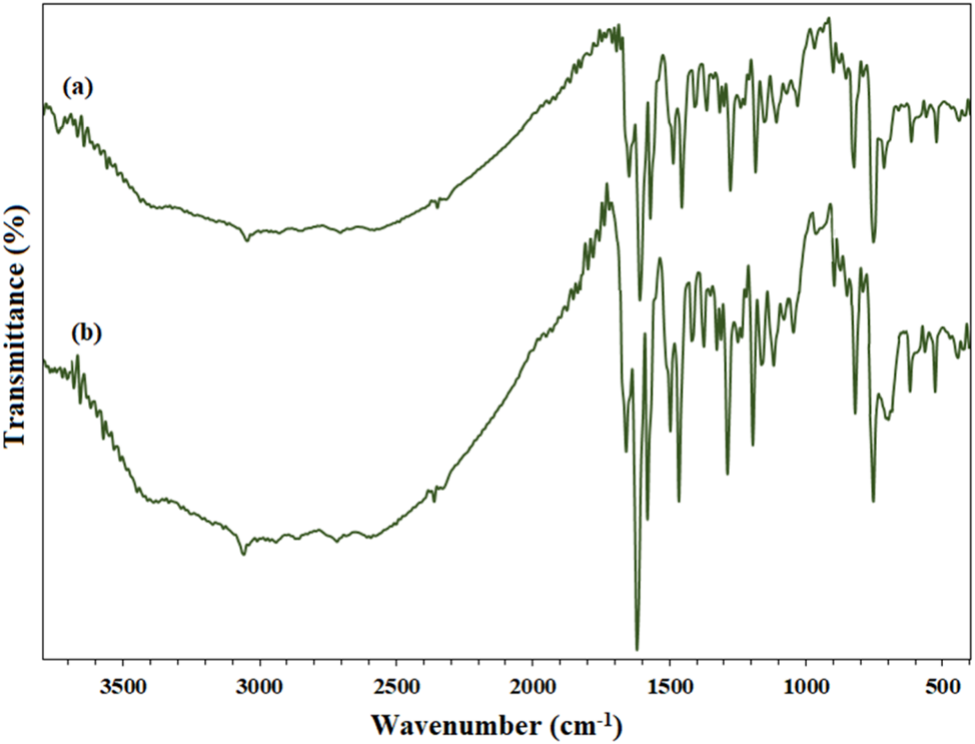
FTIR spectrum of the (a) freshly prepared PBA^30^ and (b) recycled PBA after 7th recycle in the reaction of benzyl alcohol to benzoic acid at ambient temperature.

In addition, the recovered PBA (after the 7th cycle) in the oxidation reaction of benzyl alcohol to benzoic acid was also analyzed by FTIR and compared with the spectrum of freshly prepared PBA. The FTIR analysis also confirmed the stability of the PBA structure under the reaction conditions and, as shown in Fig. 4, the recovered FTIR spectrum is quite similar to that of the freshly prepared sample.

These results prove that PBA does not become inactive in successive cycles and can be reused after recovery while maintaining its original properties. This high stability of PBA is due to its polymer structure, which contains hard benzene groups and provides its durability in successive cycles. In addition, the only active functional groups in the PBA structure are carboxylic acid groups, which are only capable of oxidation to highly acidic (and unstable) groups in the presence of an oxidant such as hydrogen peroxide, so that after conversion to highly acidic groups and undergoing oxidation and epoxidation (oxygen transfer) reactions, they return to their original structure.

### 3.5. Comparison with the literature reports

In order to highlight the advantages of the catalytic system developed in this study over previous studies, the reaction conditions for the oxidation of benzyl alcohol were compared with previously reported heterogeneous catalytic systems. Table 6 shows this comparison in terms of reaction time, % conversion, % selectivity, temperature and overall reaction conditions for the oxidation of benzyl alcohol to benzaldehyde in the presence of H_2_O_2_. Most of the previous reports are based on nanoparticles or transition metal-based catalytic systems, which are not only toxic and expensive, but also limit their applicability in industrial purposes. As shown in Table 6, the PBA/H_2_O_2_ catalytic system, in addition to being a low-cost catalytic system, has comparable performance to nanoparticle-based heterogeneous catalytic systems. It is also metal-free, which eliminates challenges such as metal leaching in different cycles that lead to a decrease in catalytic properties. The synthesis of the catalyst is relatively simple, which has advantages over catalytic systems that require several laborious steps.^55^ In addition, due to the use of cheap and readily available materials in the preparation of the catalyst, this catalytic system is cost-effective compared to other catalytic systems. High selectivity, recyclability for at least 9 consecutive times, biocompatibility, compatibility with a wide range of primary and secondary alcohol substrates, and olefins for selective oxidation reactions to aldehyde/carboxylic acid and olefin epoxidation, and scalability are other advantages of the PBA/H_2_O_2_ catalytic system, making it a reliable alternative for oxidation and epoxidation purposes.

**Table 6:** Comparison of the results of PBA/H_2_O_2_ with other catalytic systems reported in the literature for oxidation of benzyl alcohol to benzaldehyde

Entry	Conditions	Time (h)	Con. (%)/sel. (%)	Ref.
1	CuSO_4_/H_2_O_2_, H_2_O, 100 °C	0.25	98/71	49
2	[MoO_2_(L)(CH_3_OH)], H_2_O_2_, CH_3_CN, 60 °C	4	95/90	50
3	Fe(DS)_3_,^a^ H_2_O_2_, 90 °C	6	100/100	51
4	ZSM, H_2_O_2_, H_2_O, 100 °C	4	52/85	52
5	Au–Pd, H_2_O, MeOH, H_2_O_2_^b^, 120 °C	6	94.7/67	53
6	[TMGHA]_2_._4_H_0.6_PW, H_2_O, H_2_O_2_, 90 °C	6	97.9/93.5	54
8	PBA/H_2_O_2,_ R.T.	2	97/98	This work

a DS = dodecanesulfonate.

b Generated *in situ*.

### 3.6. Scalability of the process

Scalability of the process was studied over the oxidation of benzyl alcohol to benzaldehyde at 0 °C and to benzoic acid at ambient temperature by scale up to 20 mmol of benzyl alcohol (Table 7). As shown in Table 7, increasing the reaction scale to 50 mmol resulted in a slight increase in the reaction time to about 10 to 20 minutes. Furthermore, the decrease in the conversion percentage was not significant. It is noteworthy that the selectivity towards benzaldehyde and benzoic acid did not change compared to the results obtained at the small scale, which makes this process reliable and economical for industrial purposes. Therefore, the process is directly scalable and has the potential to be scaled up for industrial purposes.

**Table 7:** Scale-up study oxidation of benzyl alcohol to benzaldehyde and to benzoic acid in the presence of PBA/H_2_O_2_^a^

Entry	Substrate	Product	Time (min)	Conversion^b^ (%)/selectivity (%)
1^c^	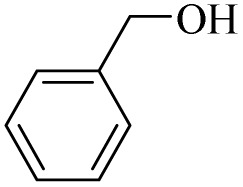	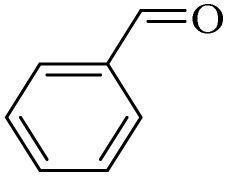	130	90/98
2^d^	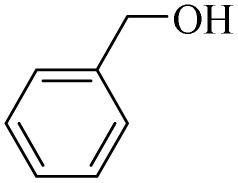	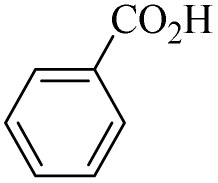	80	92/99

a The reactions were performed in a 100 mL round bottom flask and the reactions were magnetically stirred.

b GC yield. Internal standard = anisole.

c Reaction conditions: benzyl alcohol (20.0 mmol), H_2_O_2_ 35% (2.0 mL), PBA (5.0 g), 0 °C.

d Reaction conditions: benzyl alcohol (20.0 mmol), H_2_O_2_ 35% (2.0 mL), PBA (5.0 g), 26 °C.

## 4. Conclusion

In conclusion, a mild, metal free and selective method has been developed for the alcohol oxidation and olefin epoxidation. Mechanistic studies of these processes showed that the PBA generates reactive radicals in the presence of H_2_O_2_ (derived from PPBA) at room temperature, which completes the oxidation reaction of the alcohol to the carboxylic acid. At 0 °C, radical production stops and the oxidation of the alcohol proceeds through a non-radical mechanism, only up to the oxidation step to the aldehyde. Similar results were also observed for the epoxidation of olefins, but at room temperature, with the generation of radicals, the selectivity towards the epoxide decreases. PBA (or PPBA generated *in situ*) has a catalytic role in oxidation and epoxidation reactions and was capable of being recycled for at least 7 consecutive times without any loss of properties. The absence of metal in the catalyst eliminates challenges such as metal leaching, metal poisoning during the reaction, and its oxidation to inactive species. Biocompatibility, cost-effectiveness, recyclability, ease of synthesis, easy recovery, compatibility with a wide range of alcoholic and olefinic substrates, and high selectivity towards aldehyde, acid, and epoxide products are among the advantages of the PBA/H_2_O_2_ catalytic system, which makes it an effective and reliable alternative to other methods developed for oxidation purposes.

## Data availability

The authors confirm that the data supporting the findings of this study are available within the article.

## Author contributions

M. K.: conceptualization, data curation, methodology, writing original draft, writing review and editing, supervision; B. M.: data curation, formal analysis, writing review and editing, investigation. All authors reviewed the manuscript.

## Conflicts of interest

The authors declare that they have no known competing financial interests or personal relationships that could have influenced the work reported in this paper.
